# The safety of maxillary sinus floor elevation and the accuracy of implant placement using dynamic navigation

**DOI:** 10.1371/journal.pone.0304091

**Published:** 2024-05-23

**Authors:** Miaomiao Yang, Yongqing Ma, Wenli Han, Zhe Qu

**Affiliations:** 1 Department of Implantation, Dalian Stomatological Hosipital, Dalian City, Liaoning Province, China; 2 Department of Oral and Maxillofacial Surgery, Dalian Stomatological Hosipital, Dalian City, Liaoning Province, China; 3 Radiological department Dalian Stomatological Hosipital, Dalian City, Liaoning Province, China; Justus Liebig University Giessen, GERMANY

## Abstract

**Objective:**

To date, it remains a challenge to conduct maxillary sinus floor elevation (MSFE) owing to heterogeneity of anatomical structures and limited operative visibility of the maxillary sinus. The aim of this study is to investigate the safety of MSFE and the accuracy of implant placement using dynamic navigation.

**Methods:**

Forty-two implants were placed in thirty-five patients requiring implantation in posterior maxilla with dynamic navigation. They were assigned to either lateral window sinus floor elevation (LWSFE) group (n = 22) or transcrestal sinus floor elevation (TSFE) group (n = 20) according to the residual alveolar bone height (RBH). Platform deviation, apex deviation and angular deviation between actual and planned implant placement were measured in precision evaluation software. Three deviations of two groups were compared via SPSS 22.0 software.

**Results:**

Neither accidental bleeding nor perforation of Schneiderian membrane occurred in any patients. The actual window position of LWSFE was consistent with the preoperative design. There were no significant differences in platform, apex and angular deviations between the two groups (P > 0.05).

**Conclusion:**

In this study the dynamic navigation harvested clinically acceptable safety of MSFE and accuracy for implant placement in posterior maxillary region. The dynamic navigation would provide the clinician with assistance in achieving precise preoperative planning and reducing complications in surgical procedures. The granular bone grafts used in the LWSFE did not significantly affection on the accuracy of the simultaneous implant placement under the guidance of dynamic navigation.

## Introduction

Maxillary sinus floor elevation (MSFE) is a commonly used bone augmentation method when residual alveolar bone height (RBH) is insufficient in the maxillary posterior region, including lateral window sinus floor elevation (LWSFE) and transcrestal sinus floor elevation (TSFE). Many previous clinical studies have demonstrated their reliability and effectiveness in bone augmentation. However, due to the anatomic variation of maxillary sinus [1–3] and surgical limited visibility, there are risks such as the Schneiderian membrane perforation [4–6], alveolar antral artery bleeding [7,8] and even injury of adjacent teeth, which remained challenging to detect and manage during or after the surgery. Traditionally, the success of MSFE mainly depends on the experience of the surgeon. Therefore, it is necessary to develop alternative methods or supplementary measures that could effectively minimized complications and ensure the safety of surgery.

Dynamic navigation (DN) has been applied in implant surgery as an effectively preventative measure to reduce the deviation of implant placement [9,10]. DN can consistently track the 3D real-time position of the implant drills and sinus floor combined with imported Cone-beam computed tomography (CBCT) data, enable safe and accuracy location of the entry point and visualize implant placement as the planned position. The literature confirmed that implant placement with DN had not only similar deviations to surgical guide plates, but also the advantage of optimizing the procedure in real-time and enabling intraoperative, situation-related changes [11].

In recent years, there have been increasing investigations on accuracy of DN. However, there are few evaluations on its safety and accuracy in MSFE, and the impact of bone graft materials used in LWSFE on the accuracy of implant placement using DN remains unclear. Therefore, this study sought to explore the above mentioned issues and provide clinical evidence for the application of DN on MSFE in maxillary posterior dental implant surgery.

## Materials and methods

### Recruitment of the volunteers

This prospective study was approved by Ethics Committee of Dalian Stomatological Hospital (Approval number: DLKQLL202107). Between May 1, 2022 and September 30, 2023, we planed to recruit 20–40 volunteers who sought treatment for missing teeth. All volunteers submitted written informed consents to participate in this study and agreed to use their medical records for publication.

Inclusion criteria: (1) require dental implants and accept the risks involved; (2) implantation located only in unilateral maxillary posterior region; (3) insert 1–2 implants per patient; (4) agree to perform implant surgery with DN; (5) sufficient bone width; (6) the RBH was ≥ 3 mm and < 10 mm; (6) good compliance; (7) good oral hygiene care and healthy gums. Exclusion criteria: (1) limited ability to open mouth; (2) uncontrollable systemic disease; (3) history of medications and therapies known to affect bone metabolism, tissue regeneration and repair (such as glucocorticosteroids, bisphosphonates, radiation therapy, etc.); (4) suffering from periodontal disease but refusing treatment; (5) existing loosen teeth in maxillae; (6) obvious infection and inflammation of adjacent teeth; (7) having metal filling and crown on adjacent teeth; (8) maxillary sinusitis; (9) immediate implanting; (10) alcohol and/or smoke abuse.

### Experimental groups

The patients were divided into 2 groups according to the RBH. The patients in group A (3mm ≤ RBH < 6mm) received LWSFE and simultaneous implant placement, while those in group B (6mm ≤ RBH < 10mm) underwent TSFE and implant placement at the same time.

The sample size was calculate by G* Power Software (Version 3.1). A minimum sample size for each group was 17 (α = 0.05, Power = 0.08, Effect size = 1). We decided to insert 20 implants of each group at least.

### Preoperative preparation

Preoperative preparation was conducted by a trained physician familiar with DN, and all surgeries were performed by a single surgeon with 5 years of experience in computer-aided implantation surgery and 15 years of experience in free-hand implant surgery.

**Fig 1:**
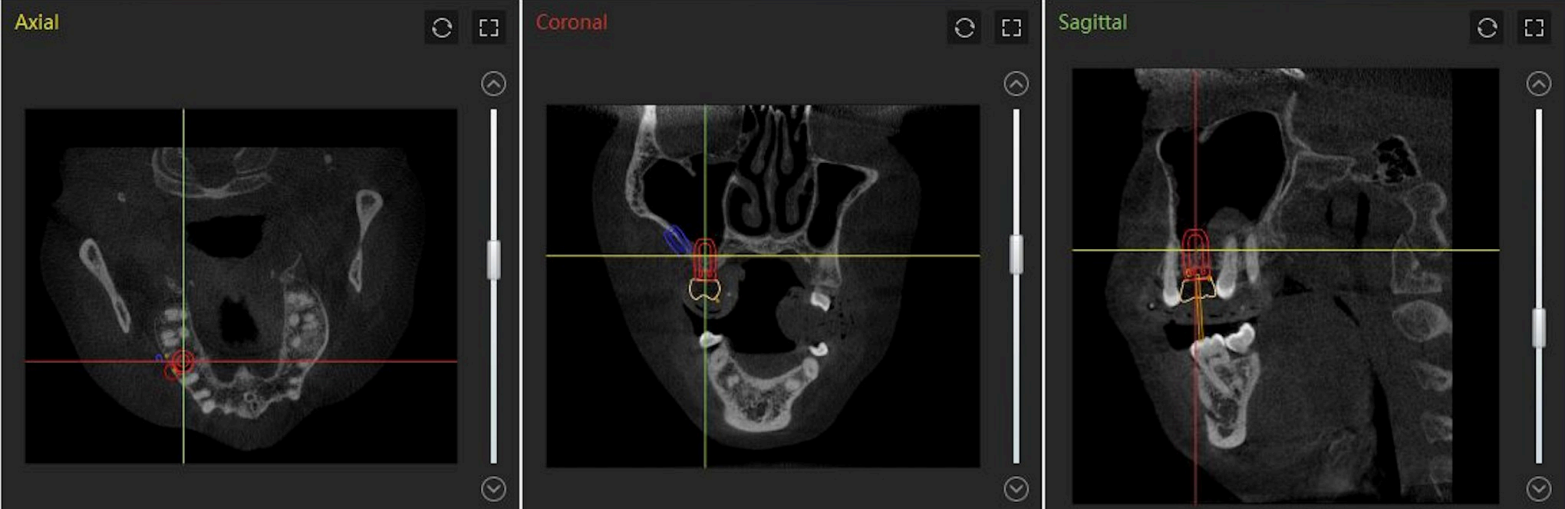
The preoperative design of virtual crown and implant placement. The Virtual crown and implant for a right maxillary first molar as planned in the dynamic navigation software.

**Fig 2:**
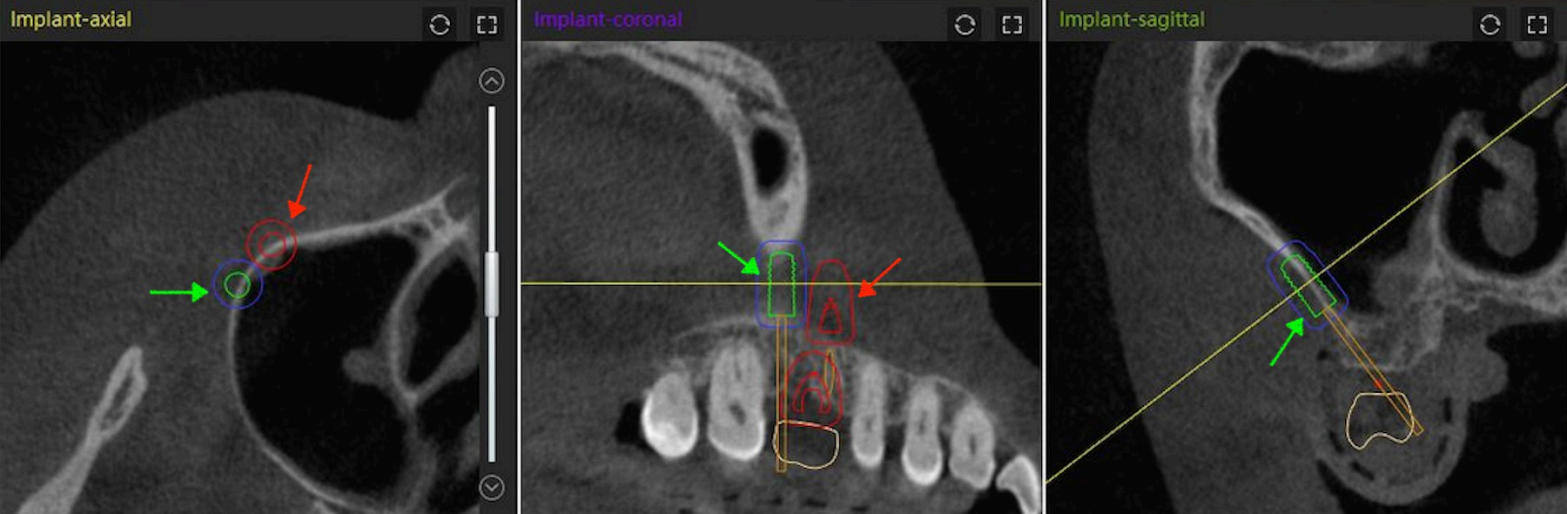
The position of the lateral window was marked. **Two implants were positioned on the surface of the sinus wall as the borders of lateral sinus window osteotomy.** The red arrow pointed mesial border; The green arrow pointed distal border.

**Fig 3:**
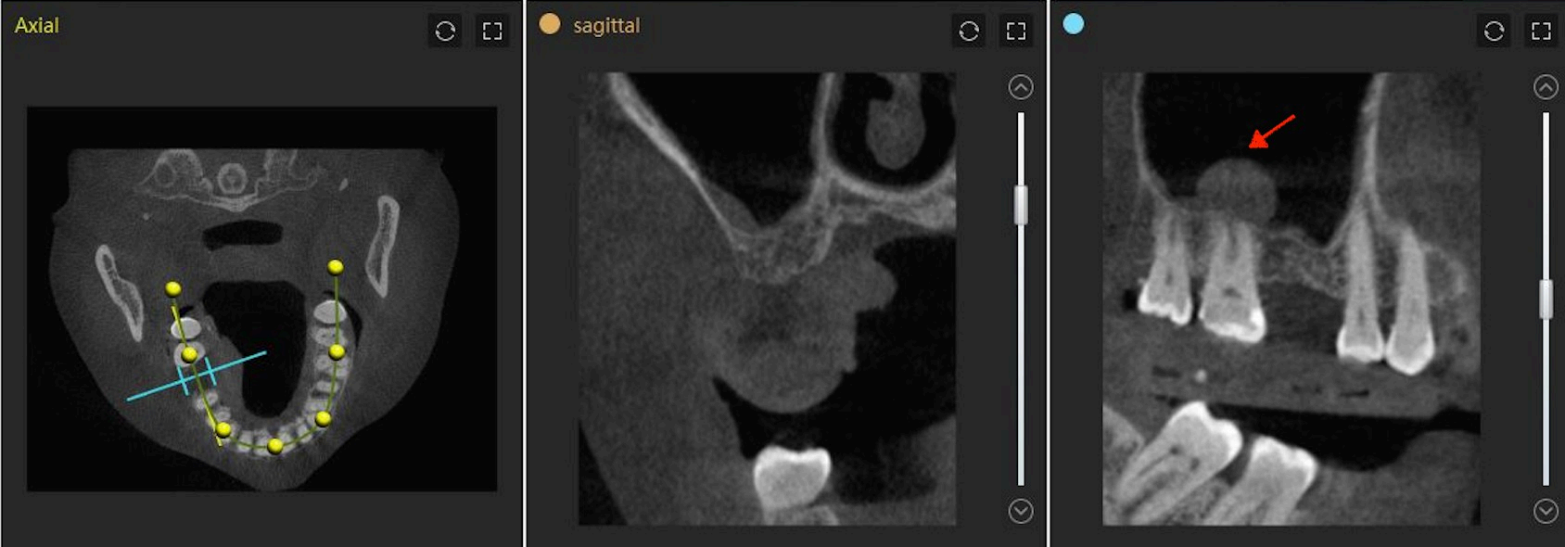
The special anatomical structures was inspected. The view of the cyst, wall thickness and Schneiderian membrane in the dynamic navigation software.

The U-shaped tube for registration was placed in the surgical sites with the polyetherimpression material(3M ESPE, Germany), and the yarn rolls were gnawed on the contralateral side to separate the U-shaped tube from the opposite teeth. Cone beam computed tomography (CBCT) scans were taken by NewTom VGi (NewTom, Italy) with a voxel size of 0.150 mm, tube voltage of 110 kV, current of 3.00 mA, and exposure time of 3.5 s, and the data were saved in DICOM file. The data was imported into the dental implant dynamic navigation system software (Dcarer, China) for preoperative planning virtualization of crown and implant placement (Fig 1). We selected the tissue level implants or the bone level tapered implants. The tissue level implants had a polished collar height of 1.8mm, a length of 10mm, and a diameter of 4.1mm or 4.8mm. The bone level tapered implants had a length of 10mm and a diameter of 4.1mm or 4.8mm. In group A, the position of the lateral window (Fig 2) was marked with two virtual implants. The sinus septa, alveolar antral artery or cyst (Fig 3) that may affect LWSFE were also marked. The patients were gargled with 0.2% compound chlorhexidine rinse in 15 minutes before surgery. The U-shaped tube and retention device for reference plate was immersed in 75% alcohol for 20 minutes.

### Surgical procedure

**Fig 4:**
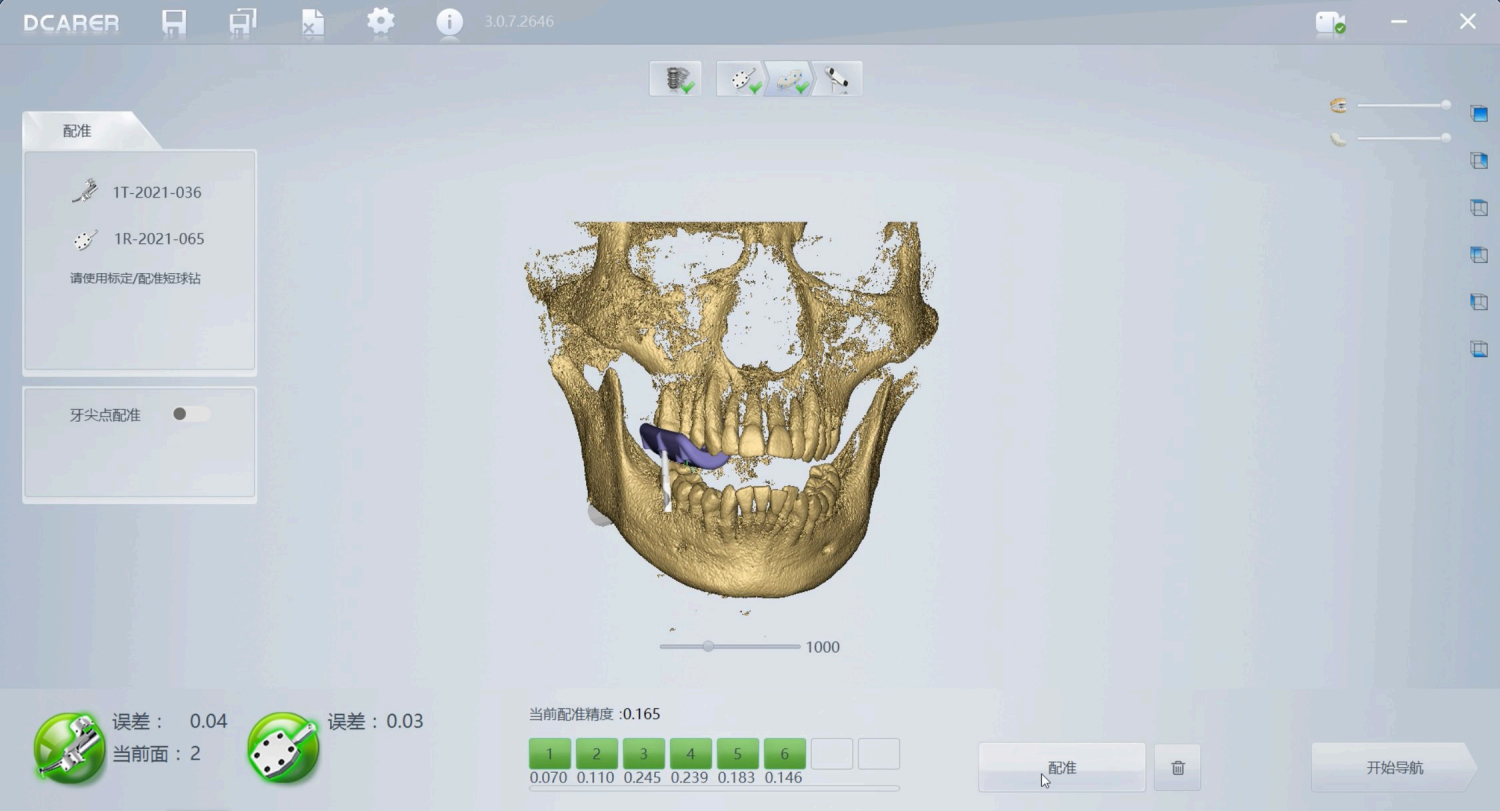
The procedure of registration in the dynamic navigation. When the registration error was less than 0.3, the dynamic navigation software entered the surgical navigation program.

**Fig 5:**
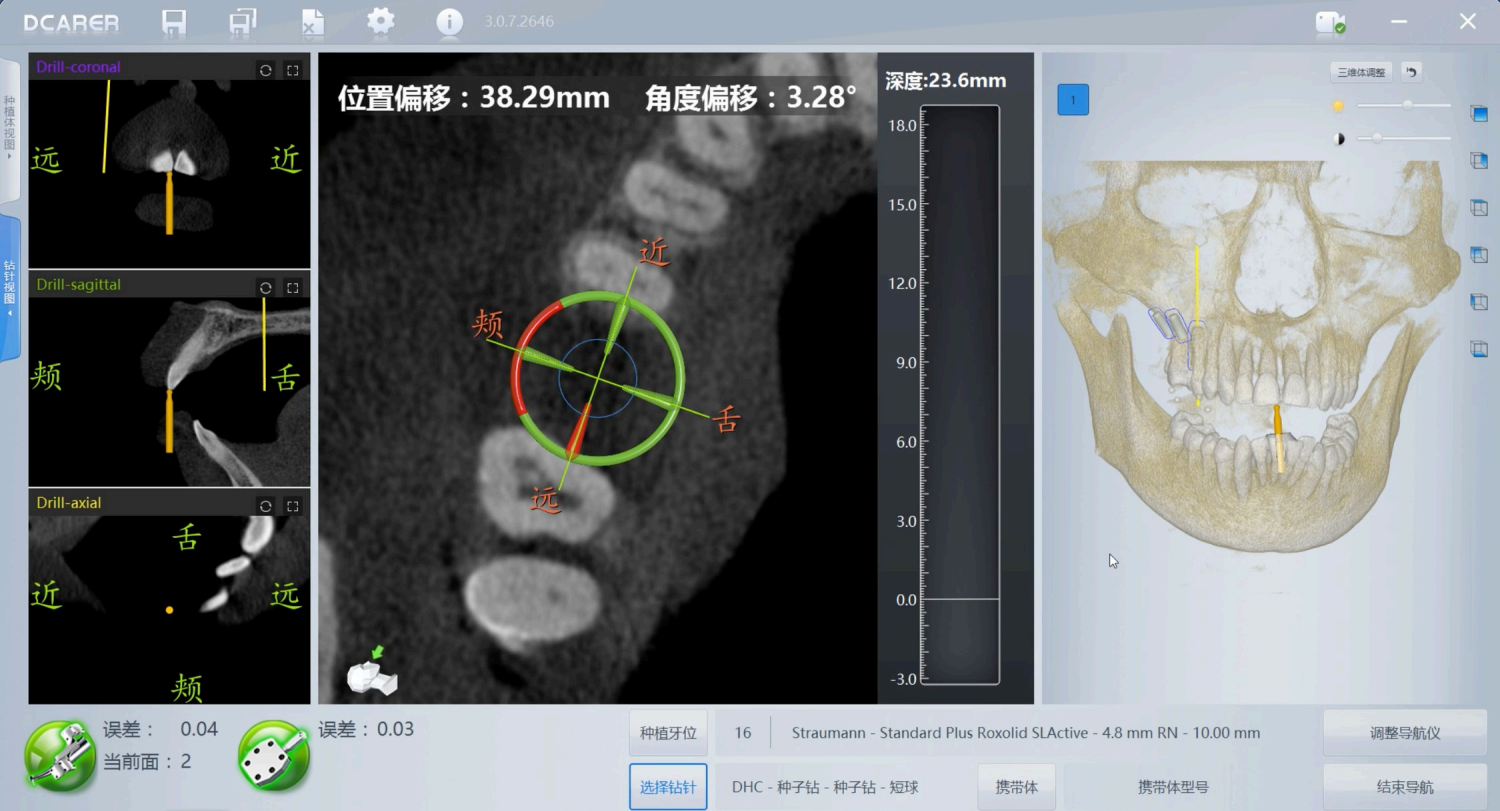
The accuracy of dynamic navigation system was tested. The short round drill was placed on the cusp of teeth in order to test the accuracy of dynamic navigation system.

**Fig 6:**
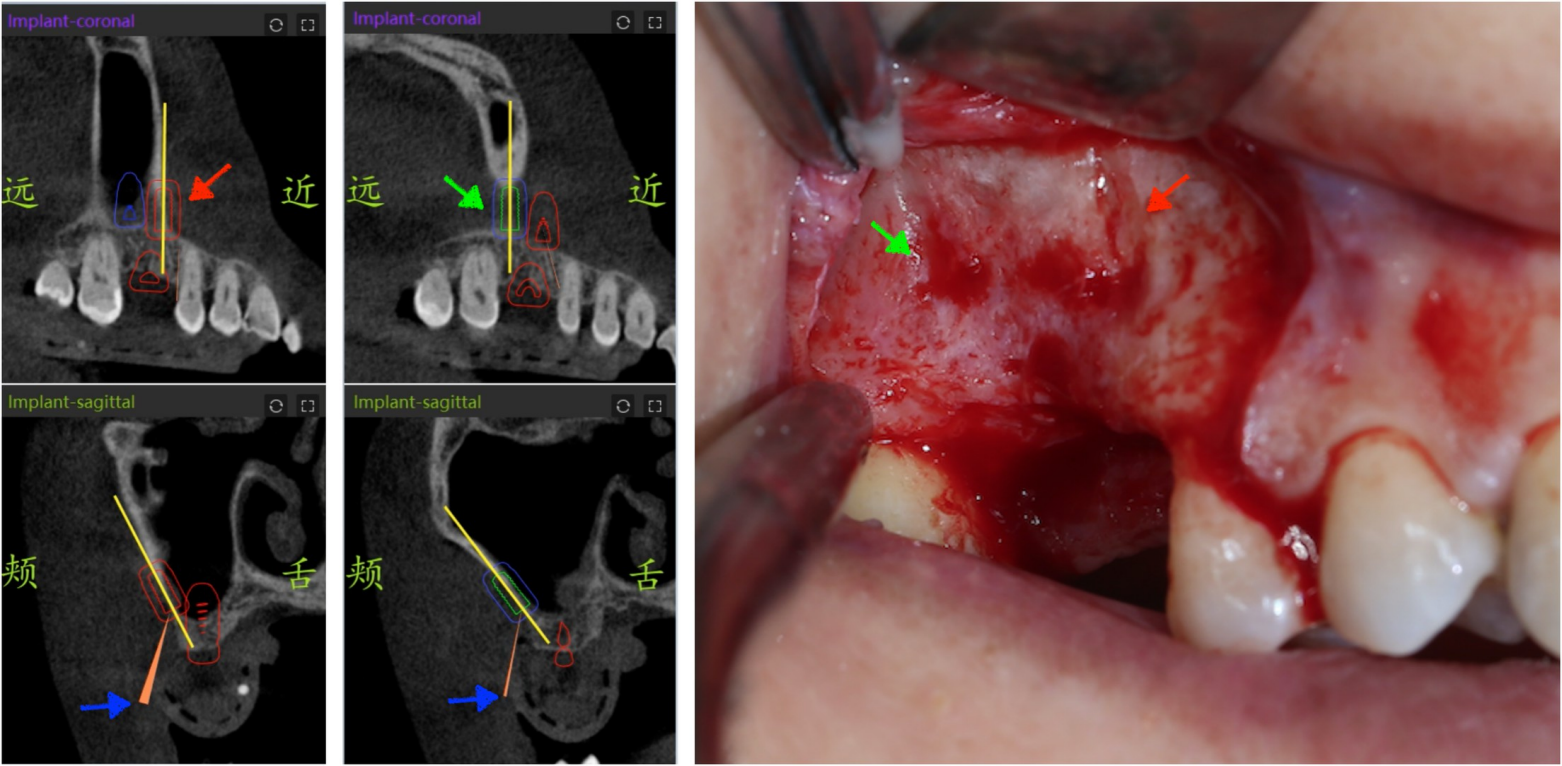
During operation the cutting lines were made guided by dynamic navigation. The red arrow pointed mesial border; The green arrow pointed distal border; The blue arrow pointed the lines representing virtual drill.

**Fig 7:**
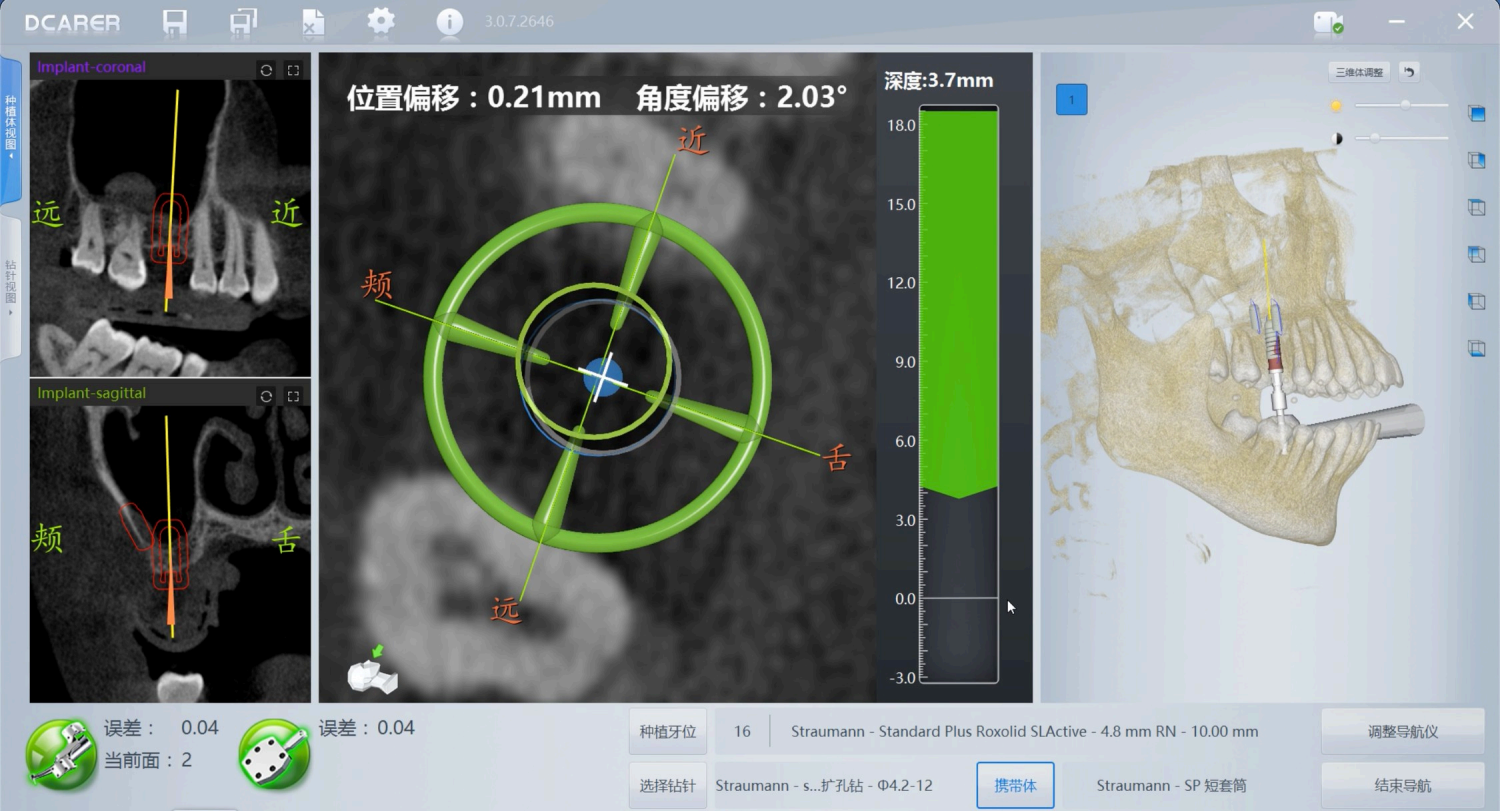
The implant was inserted under the direct view of the dynamic navigation software.

Calibration was performed for dynamic navigation instruments with handpiece locator and reference plate. The long and short drills were installed in turn to be positioned to the hemispherical groove on the reference device for calibration. The U-shaped tube was reset in the patient’s mouth and its stability was checked. The reference plate and its retention device were connected, and were fixed on the contralateral maxillary dentition with the temporary crown materials (DMG, Germany). When the registration error was less than 0.3, the navigation software could enter the surgical navigation program through the registration procedure (Fig 4). The U-shaped tube was then removed after registration and the accuracy was checked by placed the short round drill on the cusp of teeth (Fig 5). In group A, under the guidance of DN the mesial and distal boundaries of the lateral window were created on the surface of the bone by tracing the planned implants. A 2.3mm diameter round bur was used to complete the whole contour of the lateral window (Fig 6). The surgeon was able to obtain a real-time 3D visualization of the anatomic structures, such as sinus wall, alveolar antral artery, sinus septa or cyst. The surgeon was simultaneously able to watching the bur closing to the Schneiderian membrane in both virtual and real setting. A safer piezoelectric device was used to complete osteotomy when almost reaching the membrane. The Schneiderian membrane was stripped and lifted carefully. The granular bone grafts (Bio-oss, Geistlich Pharma Inc, Wolhusen Switzerland) were placed through lateral window between Schneiderian membrane and sinus floor. The implants were placed simultaneously (Fig 7). In group B, the burs were operated to reach a distance 1mm or less from the lowest point of maxillary sinus floor. The position of each burs was visible via DN software. Osteotome with matching implant diameter was used to make the sinus floor green-stick fracturing with light malleting. Bone graft material has been used when the RBH was less than 8mm. The bone graft material (Bio-Collagen, Geistlich Pharma Inc, Wolhusen Switzerland) was added upward malleting osteotome for lifting the sinus floor and membrane simultaneously. The implant was placed into designed position with DN. All implants (Straumann, Waldenburg, Switzerland) were placed according to the preoperative planned position and virtual models.

### Postoperative examination and treatment

All patients were assigned to take CBCT immediately after surgery (the same equipment), and antibiotics were prescribed for 3–5 days to prevent infection. Stitches were removed 10 days after surgery.

### Accuracy assessment of dental implant placement

The preoperative design data and the postoperative CBCT image data of implants were imported into the Dcarer dynamic navigation accuracy verification software, then the planned and the actual implant images were matched and analyzed by a blinded expert for deviation measurement. Three primary deviations were measured and recorded as follows:

Platform deviation: The linear displacement between the planned and the actual implants measuring at the centre point of implant platform.

Apex deviation: The linear displacement between the planned and the actual implants measuring at the centre point of implant apex.

Angular deviation: The angle between the central axis of the planned and actual implant.

### Statistical analysis

Statistical analysis was performed by one independent investigator using IBM SPSS Statistical Software Version 22 (IBM, USA). The data were analyzed using the independent-sample t-tests if values conformed to the normal distribution, and the Mann-whitney U test was performed if they did not. The implant platform deviation, apex deviation and angular deviation were compared between the two groups. P value less than 0.05 was regarded as significant difference.

## Results

### Demographic and clinical characteristics of the study population

A total of 42 implants were performed in 35 patients (18 females and 17 males). The age of patients ranged from 27 years old to 74 years old, and the average age was (46.69 ± 13.00) years old. The average RBH of Group A was (4.36 ± 0.95) mm, the average RBH of Group B was (8.29 ± 1.21) mm. The basic characteristic was shown in Table 1.

**Table 1:** Demographic and clinical characteristics of the study population.

	Group A	Group B
Patients	17	18
Implants	22	20
Gender(n)		
male	8	9
female	9	9
Implant location (n)		
Left side	9	12
Right side	8	8
The 1st premolar	1	0
The 2^nd^ premolar	3	8
The 1st molar	16	12
The 2^nd^ molar	2	0

### Complications

Of the 35 maxillary sinuses of all patients, there were 4 maxillary sinus septa, 3 alveolar antral arteries, 3 maxillary sinus cysts and 8 oblique sinus floors. No patients suffered Schneiderian membrane perforation caused by drills or unusual bleeding due to the injuries of alveolar antral arteries in operation. Only one patient experienced perforation while the membrane was unusually thin, and we managed properly without affecting the surgical process.

In Group A, there were different degrees of swelling on the surgical sides, lasting 3–7 days, different degrees of pain lasting 1–3 days, and no bleeding after operation. In Group B, there was no obvious swelling, pain, bleeding and vertigo. No patients complained with infection, loosening or dropping of implants during the healing period, except loosening abutments in 3 sites which were reset in time. Patients in both groups were able to tolerate varied degrees of postoperative response. CBCT examinations of both groups were performed the day after operation and there were no significant abnormalities.

### The accuracy of dental implant placement

All implants included in this study were placed under the guidance of DN. The results of accuracy verification showed that in Group A and B, the minimum and maximum deviation for platform was 0.343 mm and1.630 mm, for apex was 0.225 mm and 1.911 mm, and for angular deviation was 0.230° and 3.675°. The average platform, apex and angular deviation in Group A were (1.003 ± 0.292) mm, (0.997 ± 0.409) mm and (1.542 ± 0.845°respectively, and those in group B were (0.990 ± 0.402)mm, (1.066 ± 0.496)mm, and (1.580 ± 0.758°. The results showed that group A exhibited more platform deviation than group B, but less apex and angular deviation than group B. Anyway, there was no statistically significant difference between the two groups.

## Discussion

Accumulating literature and clinical practice have proved that MSFE is an effective protocol to solve the problem of insufficient bone height in the posterior maxillary region, including lateral window and transcrestral technique [12,13]. However, implantation in the maxillary posterior region is always a challenge for surgeons due to its special anatomical structures in the maxillary sinus. Due to the discrepancy in shapes of the sinus, bone thickness, the existence of the sinus septum, alveolar antral artery or cysts, the incidence of Schneiderian membrane perforation and accidental bleeding are inevitable [14,15]. Kang et al. believed that 10–30% of lateral window osteotomy would be affected by alveolar antral artery [16]. Despite the advancement of CBCT in the last decades, it is still a challenge for surgeons to precisely position vessels and bony septa in lateral sinus wall and perform the fenestration as designed position. Likewise, the drills should be terminated at a distance of less than 1mm from the sinus floor during the TSFE. Too thick residual bone would make it difficult for the elevation of the sinus floor and the Schneiderian membrane. On the opposite, too thin residual bone would increase the risk of Schneiderian membrane perforation. Therefore, it is essential for surgeons to track and adjust the position of the implant drill in real-time 3D visuals.

In recent years, the DN has been increasingly used to assist surgeons to improve the precision of surgery [9,10]. Studies have indicated that the accuracy of DN is similar to that of a static guide in assisting implant placement [17–19], and both of which are better than that of the free hand [20–23]. Currently, few reports about the application of DN in MSFE have been found after a throughout search [24,25]. Wu et al reported the application of DN in the surgery of TSFE [24]. Bishbish et al conducted a pilot application of DN in LWSFE [25]. Bone grafts applied during MSFE, especially in LWSFE, however, whether they would affect the accuracy of implant placement under DN has not been reported in the literature.

The perforation of the Schneiderian membrane commonly occurs while it is lifted. This risk is related to the proficiency of the surgeon and the anatomical structures of the maxillary sinus. In this study, the surgeon designed the optimal lateral window position in DN software, and they were able to track the burs and monitor the special anatomical structures of the maxillary sinus in the operation, such as the morphology of maxillary sinus floor, sinus wall and sinus septa. As a result the risk of Schneiderian membrane perforation might be reduced. In Group B the burs guided by DN were stopping at 1 mm or less from the lowest point of maxillary sinus floor, the remaining sinus floor was thin enough to be lifted up easily, thus patients would not experience obvious vertigo [26].

In a recent systematic review on the accuracy of implantation via DN, Yu et al [27] retrospected clinical studies in the past 10 years and reported the mean values of global platform deviation, global apical deviation and angular deviation as 1.07 mm (95% CI: 0.96–1.17), 1.27 mm (95% CI: 1.06–1.47) and 3.43° (95% CI: 2.94–3.93), respectively. In this study the platform deviation and apical deviation were consistent with the previous studies except smaller angular deviations. It was reported that the accuracy of implantation might be influenced by the uneven shape of alveolar crest.Therefore, in our study, cases with insufficient horizontal bone width were excluded, and only cases requiring vertical bone augmentation were included, aiming to eliminate the influence on the accuracy of implantation derived from the shape of alveolar crest. And it might be also the reason for the smaller angular deviation in this study.

Wu et al’s research showed that the angular deviation of maxillary premolar sites was smaller than that of molar sites, and the difference between which was statistically significant [25]. Apparently, the results of our study are not the same as those of Wu’s. In detail, group A included 4 implants in the premolar area and 18 implants in the molar region. In group B, there were 8 premolar implants and 12 molar implants, which were relatively anterior compared with group A. But the discrepancy in implant sites did not result in significant differences in the accuracy of implant. The maximum linear deviation in this study was 1.603 mm, which suggested that we should ensure appropriate safety distance while performing implants guided by DN. Especially in TSFE, clinicians should combine clinical experience with the real-time visual image of DN to avoid drilling through the maxillary sinus floor.

A study by Ozan on surgical guides showed that the lower bone density values might lead to the greater angular deviations [28]. This result might be caused by the fact that surgical guides were only used to guide drilling and not inserting implants. Ozan pointed that it was important to use surgical guides throughout the entire process to prevent additional angular deviation when the density of alveolar bone was low. In this study, the cortical bone in the maxillary posterior region was thin, the cancellous bone was loose, and the bone density was low. Although bone grafts used in LWSFE might have more radiographic density than the pristine bone, they were actually composed of xenograft particles, the bone grafts might be more loose than the natural bone. In our study the natural bone height of Group A was lower than Group B. The length of the implant inserted into the granular bone grafts in Group A was approximately grater than 4 mm. There were no significant differences in platform, apex and angular deviations between the two groups. This results indicated that when both drilling and inserting implant were guided by DN, the accuracy of dental implant placement of Group A had not been affected by the granular bone grafts compare to Group B.

DN has been used the guidance of dental implant placement for several years. We can plan accurately and execute effectively with DN. But when we use DN, we still need to pay attention to some points. All devices installed in the mouth need to be kept stable. The U-shaped tube for registration need support from adjacent teeth. Therefore, we need to ensure that adjacent teeth are not loose. If the consecutive missing teeth are more or located on the free ends of the posterior region, the stability of the U-shaped tube may be affected. We can use titanium nails as registration devices instead of U-shaped tube. In this study, two patients had their reference plates loosening before the surgery began. We promptly fixed again, and the registration was re executed. During the operation is performing, we often need to check the stability of the reference plate. The application of DN is to some extent limited by metal fillings or crowns. The artifacts generated by metals in radiographic images may affect the recognition and matching of U-shaped tube. If there are too many metal filling or crowns, the static surgical guides may be a better choice instead of DN. During the process of using DN to guide surgery, we should always pay attention to whether the actual situation is consistent with the images in the software.

## Conclusion

In summary, with the guidance of DN, the LWSFE and TSFE could be performed safely and effectively, which might be a promising method to reduce relevant complications. When performing implantation in maxillary posterior region with the assistance of DN, the deviation between the achieved and designed positions was not significant and fulfilled with the clinical requirements. The granular bone grafts used in the LWSFE had no significant affection on the accuracy of the simultaneous implant placement when the full procedures inserted were under the guidance of DN.

## Supporting information

S1 Data Minimal data set. (XLSX)
